# Evidence-based Clinical Decision Support Systems for the prediction and detection of three disease states in critical care: A systematic literature review

**DOI:** 10.12688/f1000research.20498.2

**Published:** 2019-11-27

**Authors:** Goran Medic, Melodi Kosaner Kließ, Louis Atallah, Jochen Weichert, Saswat Panda, Maarten Postma, Amer EL-Kerdi

**Affiliations:** 1 Health Economics, Philips, Eindhoven, Noord-Brabant, 5621JG, The Netherlands; 2 Department of Pharmacy, Unit of PharmacoTherapy, -Epidemiology & -Economics, University of Groningen, Groningen, 9700 AB, The Netherlands; 3 Global Market Access Solutions Sàrl, St-Prex, 1162, Switzerland; 4 Philips, Cambridge, MA, 02141, USA; 5 Department of Health Sciences, University Medical Centre Groningen, University of Groningen, Groningen, 9700 AB, The Netherlands; 6 Department of Economics, Econometrics & Finance, University of Groningen, Groningen, 9700 AB, The Netherlands

**Keywords:** sepsis, hemodynamic instability, respiratory distress, infection, machine learning, clinical trials, critical care.

## Abstract

**Background: **Clinical decision support (CDS) systems have emerged as tools providing intelligent decision making to address challenges of critical care. CDS systems can be based on existing guidelines or best practices; and can also utilize machine learning to provide a diagnosis, recommendation, or therapy course.

**Methods: **This research aimed to identify evidence-based study designs and outcome measures to determine the clinical effectiveness of clinical decision support systems in the detection and prediction of hemodynamic instability, respiratory distress, and infection within critical care settings. PubMed, ClinicalTrials.gov and Cochrane Database of Systematic Reviews were systematically searched to identify primary research published in English between 2013 and 2018. Studies conducted in the USA, Canada, UK, Germany and France with more than 10 participants per arm were included.

**Results: **In studies on hemodynamic instability, the prediction and management of septic shock were the most researched topics followed by the early prediction of heart failure. For respiratory distress, the most popular topics were pneumonia detection and prediction followed by pulmonary embolisms. Given the importance of imaging and clinical notes, this area combined Machine Learning with image analysis and natural language processing. In studies on infection, the most researched areas were the detection, prediction, and management of sepsis, surgical site infections, as well as acute kidney injury. Overall, a variety of Machine Learning algorithms were utilized frequently, particularly support vector machines, boosting techniques, random forest classifiers and neural networks. Sensitivity, specificity, and ROC AUC were the most frequently reported performance measures.

**Conclusion: **This review showed an increasing use of Machine Learning for CDS in all three areas. Large datasets are required for training these algorithms; making it imperative to appropriately address, challenges such as class imbalance, correct labelling of data and missing data. Recommendations are formulated for the development and successful adoption of CDS systems.

## Introduction

Critical care, including intensive and emergency care, is the most expensive and human resource intensive area of in-hospital care. Despite having the most technologically advanced devices, it is the area associated with the highest morbidity and mortality rates ^1^. Decision-making for clinical teams in this area is complex due to variability in procedures and data-overload from the plethora of existing devices. In fact, misdiagnosis in the intensive care unit (ICU) is 50% more common than other areas ^2^, and errors, especially medication errors which account for 78% of serious medication errors ^3^, can have a long lasting effect even after patients are discharged.

Computerized decision support (CDS) systems have emerged as tools providing intelligent decision making based on patient data to address many of the challenges of critical care. CDS systems can be based on existing guidelines or best practices; and can also utilize machine learning as a means of compiling several data inputs to provide a diagnosis, recommendation, or therapy course. CDS systems can improve medication safety by providing recommendations relating to dosing ^4– 6^, administration frequencies ^5^, medication discontinuation ^6^ and medication avoidance ^5^. Moreover, these novel systems can improve the quality of prescribing decisions by triggering alerts or warning messages on drug duplication, contraindications, drug interaction errors ^7^, side-effects and inappropriate medication orders ^5^. CDS system notifications can be applied during the prescribing, administering or monitoring stages to detect and prevent medication errors ^8^. These systems can also target patients to facilitate shared decision-making to empower as well as to motivate them ^9– 11^. The need for such systems stems from hospitals having to deal with strict guidelines to improve outcomes, document care cycles (raising the need for administrative tasks) and reduce readmissions. This is combined with the need to cope with financial constraints, such as staff shortages and increased pressure to reduce the length of stay ^12, 13^.

Strategies for bringing CDS to clinics have been the topic of several workshops, conferences and focus groups ^14^. Factors for success in designing CDS include providing measurable value, producing actionable insights, delivering information to the user at the right time, and demonstrating good usability principles ^14^.

Early warning systems (EWS) are CDS systems designed for initial assessment and identification of patients at risk of deterioration in in-patient ward areas ^15– 17^. These systems have shown that they can enable caregivers and rapid response teams to respond earlier – in time to make a difference ^18^. By alerting clinicians to higher risk patients, treatments can be administered early or harmful medications can be stopped, potentially leading to improved outcomes. Early recognition and timely intervention are also critical steps for the successful management of shock ^19^, cardiorespiratory instability ^20^ and severe sepsis. In sepsis management, adequate timing of administration of antibiotics is directly associated with survival rates ^21^, and incidence, severity and duration of infections.

According to the Society of Critical Care Medicine (SCCM) ^22^, the five primary ICU admission diagnoses for adults are respiratory insufficiency/failure with ventilator support, acute myocardial infarction, intracranial hemorrhage or cerebral infarction, percutaneous cardiovascular procedures, and septicemia or severe sepsis without mechanical ventilation. SCCM also highlights other conditions involving high ICU demand such as poisoning and toxic effects of drugs, pulmonary edema and respiratory failure, heart failure and shock, cardiac arrhythmia and renal failure. Given the above, three high-impact areas were selected for the current research where early detection and treatment could impact outcomes for patients in the ICU. The first is that of hemodynamic instability, where early detection could help patients prevent deterioration into shock. The second is that of respiratory distress, affecting many ventilated patients (up to 40% are ventilated according to SCCM) ^22^. The third area selected is that of infection, with a focus on sepsis. Sepsis is the most common cause of death among critically ill patients, with occurrence rates varying from 13.6% to 39.3% ^23, 24^. All three areas are major areas of concern with relatively high prevalence in critical care having long term effects on patients.

The study focuses on both detection, which alerts the clinician to the presence of these specific conditions, as well as prediction of deterioration by alerting the clinician in advance that a patient will deteriorate into one of these disease states. The aims of this study were to perform and report a systematic review of the utilization of CDS systems in the three selected disease areas and summarize the methodological aspects of identified studies.

## Methods

### Search strategy

A systematic literature review was carried out to identify evidence-based study designs, methods and outcome measures that have been used to determine the clinical effectiveness of CDS systems in the detection and prediction of three populations representing the variety and majority of morbid conditions in a critical care setting: Shock (hemodynamic (in-)stability), respiratory distress/failure and infection/sepsis. The search strategy combined ‘intervention terms’ and ‘disease terms’ to identify primary research evaluating the diagnostic performance of CDS systems and other machine learning algorithms in three different populations of any age, sex, and race. Systematic literature reviews were also included for locating further relevant primary research. The search was conducted in MEDLINE (PubMed), ClinicalTrials.gov and Cochrane Database of Systematic Reviews (CDSR); and limited to studies published or registered between January 1, 2013 and November 8, 2018 and reported in English. Publication dates were limited to focus results on the most recent developments in this fast-evolving research domain. Another method to ensure up-to-date results was to include conference abstracts from 2017 onwards regardless of whether or not they were followed up with a detailed publication. Ongoing studies identified in the clinical trials register were also kept in the review. Study protocols identified from bibliographic databases were, however, excluded assuming that final study results would be available and identified elsewhere. The strategy employed in PubMed is provided as *Extended data*, Table 1–Table 3 ^25– 27^.

Studies conducted in US, Canada, UK, Germany or France with more than 10 subjects per arm were included. These countries were selected because they are known to be active in CDS development. The inclusion and exclusion criteria for selecting abstracts and subsequent full-text publications were based on the population, interventions, comparators, outcomes, and study design (PICOS). These criteria are listed in Table 1.

**Table 1.** Study selection criteria for the systematic literature review.

Criteria		Inclusion	Exclusion
**STUDY DESIGN**	**Abstract** **selection**	Randomized controlled trials (RCT) Observational (retrospective and prospective) studies In-hospital settings: Acute care, Intensive care unit (ICU), Emergency department (ED), Medical Surgery, General ward Geography: US, Canada, Europe	Systematic Literature Reviews or meta- analyses * Review papers, newsletters and opinion papers where treatments of interest are only discussed Methodology studies or protocols Case studies (sample size of 1 patient) Studies with less than 10 patients per arm; Conference abstracts published only as abstracts in 2013, 2014, 2015 and 2016 Geography **: All countries and regions except: US, Canada, UK, Germany, France Publications without an abstract
	**Full-text** **selection**	Randomized controlled trials (RCT) Observational (retrospective and prospective) studies In-hospital settings: Acute care, Intensive care unit (ICU), Emergency department (ED), Medical Surgery, General ward Geography **: US, Canada, UK, Germany, France Conference abstracts published only as abstracts in 2017 and 2018	Systematic Literature Reviews or meta- analyses * Review papers, newsletters and opinion papers where treatments of interest are only discussed Methodology studies or protocols Case studies (sample size of 1 patient) Studies with less than 10 patients per arm; Geography **: All countries and regions except: US, Canada, UK, Germany, France Publications published only as abstracts in 2013, 2014, 2015 and 2016 (which were not superseded by full-text publication).
**POPULATION**	**Abstract** **and full-text** **selection**	Studies that include humans only – adults, children and neonates (or (electronic) medical records) Both sexes are included Patients with or at risk of developing shock (hemodynamic (in-stability) Patients with or at risk of developing respiratory distress/failure Patients with or at risk of developing infection or sepsis Healthy people only; Healthy people and patients	*In-vitro* studies Animal studies
**TREATMENT /** **INTERVENTION**	**Abstract** **and full-text** **selection**	Artificial intelligence Machine learning (i.e. Deep learning models) Clinical decision support Computer aided detection Early Warning System	Automatic diagnosis systems (i.e. ELISA tests) Screening tests (i.e. Automated analysis of portable oximetry) Sequencing tests Mathematical models *** - which model the predictability of disease or treatment/ intervention (i.e. Modelling studies have been widely used to inform human papillomavirus vaccination policy decisions) Multivariable hierarchal logistic regression models *** (models which are based only on statistics - but there is no machine learning)
**COMPARATOR**	**Abstract** **and full-text** **selection**	All comparators	No selection will be made regarding comparator
**OUTCOMES**	**Abstract** **and full-text** **selection**	Detection and/or prediction outcomes, such as: • Sensitivity (SD) (%) • Specificity (SD) (%) • NPV (%) • PPV (%) • Likelihood ratio • Accuracy (SD) (%) • Prevalence of disease (%) • OR; 95% CI; p-value • HR; 95% CI; p-value • Median (IQR); p-value • ROC AUC For all outcomes (if reported): Measure of variability (i.e. Standard error of mean (SE), Standard deviation (SD)); measure of uncertainty (i.e. 95% CI) The outcomes should be reported in the following manner: • per arm (study group vs. control group) individually; • difference between 2 arms.	Studies not reporting detection and/or prediction outcomes Studies discussing interventions of interest, but no outcomes are reported

* Systematic Literature Reviews and (network) meta-analysis are excluded from data extraction since the pooled results cannot be used in our analysis. However, good quality (network) meta-analysis and systematic literature reviews (i.e. Cochrane reviews) will be used for cross-checking of references if the search did not omit any articles.

** If studies are conducted in multiple countries and at least 1 of the included countries is included – the study will be included in the selection.

*** Mathematical and logistic regression models – can be used to validate and evaluate Interventions of interest (that are listed as included intervention), but the texts discussing these models without any “learning potential” or artificial intelligence potential will be excluded. Therefore, these models can be the foundation of the included listed interventions but will not be included in the Data Extraction Files unless they have also machine learning or artificial intelligence or some other form of “learning potential” on top of the statistical mathematical model. Researchers will pay special attention and caution when screening these abstracts and/or full-text articles.

AUC = Area under the curve; ED = Emergency department; ELISA = Enzyme-linked immunosorbent assay; HR = Hazard ratio; ICU = Intensive care unit; IQR = interquartile range; NPV = Negative predictive value; OR = Odds ratio; PPV = Positive predictive value; RCT = Randomized controlled trial; ROC = Receiver Operating Characteristic; SD = Standard deviation; SE = Standard error; UK = United Kingdom; US = United States.

### Study selection and data extraction

Study selection and data extraction was carried out by a single reviewer (MKK or SP). In cases of uncertainty, a second, or even third reviewer, was consulted. Data extraction was performed using a standard data extraction form (DEF). Key data from each additional eligible study were extracted by recording data from original reports into the DEF. The DEF included information on study design, inclusion/exclusion criteria, sample size and characteristics, interventions, outcome measures (measures of predictability like: sensitivity, specificity, negative predictive value (NPV), positive predictive value (PPV), likelihood ratio, accuracy (percentage of correctly identified cases in relation to the whole sample), odds ratio (OR), hazard ratio (HR), median, receiver operating characteristic (ROC) area under the curve (AUC); and length of hospitalization among others).

Studies identified from the ClinicalTrials.gov registry that did not report results were also included in the extraction to give some indication of the outcomes being collected.

### Study quality appraisal

This research was not aimed at summarizing study results and assessing the relative effectiveness of CDS systems. Therefore, an appraisal of study quality was not deemed necessary.

## Results

### Shock (hemodynamic (in-)stability)

**Figure 1.**
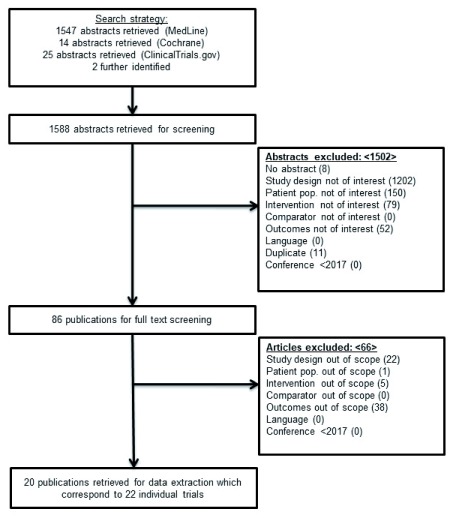
Study selection – Shock. Pop. = Population.

The search yielded 1588 hits. Screening the titles and abstracts led to 1502 being excluded. The full texts of the remaining 86 titles were obtained and assessed against the PICOS criteria. Studies were excluded due to irrelevant study design (n=22), population (n=1), intervention (n=5), and outcomes (n=38). A total of 20 studies were finally included in this systematic literature review. This included 5 trials identified from ClinicalTrials.gov. The study selection process is depicted in Figure 1.

***Study characteristics***. Of the 15 published studies, five were conducted by research groups outside the USA ^28– 32^. Ten studies were conducted in the US ^19, 33– 41^, Thirteen studies were retrospective ^19, 28– 33, 35, 37– 41^ and only two were prospective ^34, 36^. Nine studies were single-center ^28, 30, 31, 33, 37– 41^ and six studies were multi-center ^19, 29, 32, 34– 36^. Five studies were time-series ^28, 30– 32, 40^ and nine were case-series ^19, 29, 33– 35, 37– 39, 41^.

Across all studies, three had sample sizes ≤100 ^29, 30, 36^; three had sample sizes of 101–1000 ^28, 31, 32^; four studies had sample sizes of 1001–10,000 ^19, 33, 34, 37, 42^; and another five studies, four retrospective single-center studies and one multi-center, had sample sizes larger than 10,000 ^35, 38– 41^. The three largest studies included patients admitted to various wards of a specified hospital. The majority of the studies did not restrict their sample to a specific in-patient hospital setting. Five studies reported on patients in the ICU ^19, 28, 32, 40, 41^ and one study reported on patients admitted to the surgical ward ^33^.

The characteristics of the published studies are summarized in Table 2.

**Table 2.** Design aspects of published studies on shock.

Study	Study Design	Country and institution(s)	Number of patients (records)	Population/disease definition	In- patient setting	Collected data
Ghosh 2017	Retrospective time series single center	Australia University of Technology Sydney & The University of Melbourne	209	Sepsis or severe sepsis	ICU	(mean arterial pressure), heart rate, respiratory rate
Hu 2016	Retrospective case series single center	USA, Minnesota University of Minnesota	NR (8909)	NR	Surgery	EHRs
Li 2014	Retrospective case series multi-centric (3 centers)	UK, Oxford University of Oxford & Mindray	NR (67)	Ventricular flutter, fibrillation and tachycardia	NR	Electrocardiography
Mahajan 2014	Prospective case series multi-centric (4 centers)	USA University of Southern California, Mayo Clinic- Rochester, University of North Carolina, Sanger Heart & Vascular Institute & Boston Scientific	410 (908)	Ventricular fibrillation, ventricular tachycardia and other arrhythmias	NR	Electrograms
Mao 2018	Retrospective case series multi-centric (5 centers)	USA University of California, Stanford Medical Centre, Oroville Hospital, Bakersfield Heart Hospital, Cape Regional Medical Centre, Beth Israel Deaconess Medical Center	359,390	NR	various	Vital signs
Reljin 2018	Prospective case- control multi-centric (2 centers)	USA University of Connecticut, Campbell University School of Medicine, University of Massachusetts Medical School,Yale University School of Medicine & Worcester Polytechnic Institute	36 (94)	Traumatic injury, healthy controls	NR	Photoplethysmographic signals
Sideris 2016	Retrospective case series single center	USA, Los Angeles University of California	1948	Primarily heart failure	various	EHRs
Blecker 2016	Retrospective case series single center	USA, New York NewYork-Presbyterian Hospital & New York University	NR (47,119)	NR	various	EHRs
Blecker 2018	Retrospective case series single center	USA, New York New York University	NR (37229)	NR	various	EHRs
Calvert 2016	Retrospective time series single center	USA, California Dascena Inc. & University of California	29083	NR	ICU	vital signs
Donald 2018	Retrospective time series + Prospective time series multi-centric (22 centers)	Europe	173	Traumatic brain injury	ICU	Demographic, clinical and physiological data
Ebrahimzadeh 2018	Retrospective time series single center	Iran University of Tehran, Iran University of Science and Technology, University of Sheikhbahaee & Payame Noor University of North Tehran	53 (106)	Paroxysmal atrial fibrillation	NR	Electrocardiography
Potes 2017	Retrospective case series multi-centric (2 centers)	USA, California & UK, London Children`s Hospital Los Angeles, St. Mary`s Hospital, London & Philips	8022	NR	ICU	Vital signs, laboratory values, and ventilator parameters.
Henry 2015	Retrospective case series single center	USA, Maryland John Hopkins University	16234	NR	ICU	EHRs
Strodthoff 2018	Retrospective time series single center	Germany, Berlin Fraunhofer Heinrich Hertz Institute & University Medical Center Schleswig- Holstein, Kiel	200 (228)	Myocardial infarction and healthy controls	NR	Electrocardiography

USA: United States of America. UK: United Kingdom. NR: Not reported. ICU: Intensive care unit. EHR: Electronic health records.

***CDS systems***. Machine learning algorithms were developed to detect or predict septic shock ^28, 33, 35, 40, 41^, various heart arrhythmias ^29, 30, 34^, heart failure ^37– 39^, hemodynamic instability and hypovolemia ^19, 36^, myocardial infarction ^31^, as well as hypotension ^32^.

All studies, except one, trained a single algorithm. Ebrahimzadeh *et al.* 2018 ^30^ trained and compared support vector machine (SVM), instance-based and neural network models to predict paroxysmal atrial fibrillation. SVMs were the most frequently used algorithms, followed by least absolute shrinkage and selection operator (LASSO) regularization. In one study, the SVM was trained using sequential minimal optimization ^37^.

Machine learning models were trained and validated in 14 studies and subsequently tested in an independent dataset in 3 studies ^19, 35, 37^. In one study an algorithm trained to classify arrythmias was not validated but compared to physician`s manual classifications ^34^.

An overview of the investigated machine learning algorithms is presented in Table 3.

**Table 3.** Overview of the algorithms developed to detect shock.

Study	Predicted disease	Learning algorithm												
		CHMM	Decision trees	LR, LASSO regularisation	LR, not specified	SVM	kNN	RF	gradient tree boosting	Adaptive boosting	Bayesian neural network	convolutional neural network	Multilayer perceptron	mixture of expert
Ebrahimzadeh 2018	paroxysmal atrial fibrillation					✓	✓						✓	✓
Li 2014	Ventricular fibrillation and tachycardia					✓								
Mahajan 2014	heart arrhythmias					✓								
Strodthoff 2018	myocardial infarction											✓		
Sideris 2016	heart failure					✓								
Blecker 2016	heart failure			✓										
Blecker 2018	heart failure			✓										
Reljin 2018	Hypovolemia					✓								
Potes 2017	hemodynamic instability									✓				
Donald 2018	Hypotension										✓			
Ghosh 2017	septic shock	✓												
Hu 2016	septic shock			✓										
Mao 2018	septic shock								✓					
Calvert 2016	septic shock				✓									
Henry 2015	septic shock			✓										

CHMM: clustered hidden Markov model. LR: Logistic regression. SVM: Support vector machine. kNN: k nearest neighbor. RF: Random forest. Conv.: Convolutional.

***Outcome measures***. Three of the 15 papers measured a single outcome of model performance. In two studies the preferred measure was accuracy ^28, 34^; whereas in another study this was the ROC AUC. This study was large and based their algorithm on EHRs ^33^. Across all studies, accuracy was reported in about half of the instances and the ROC AUC was one of the most frequently reported outcomes.

Sensitivity and specificity were reported together in 10 studies. Blecker *et al.* 2016 ^38^ reported sensitivity together with PPV. Sensitivity and specificity were not measured in the study by Sideris *et al.* 2016 ^37^, instead model accuracy and the ROC AUC were preferred. This study was concerned with developing an alternative `comorbidity` framework based on disease and symptom diagnostic codes to cluster individuals at low to high risk of developing chronic heart failure.

PPVs were reported in six studies and accompanied with negative predictive values in two studies. These studies developed and validated machine-learning algorithms for the early detection of less investigated health conditions, these being hemodynamic instability in children ^19^ and acute decompensated heart failure ^39^. The highest number of outcome measures, including likelihood ratios, was observed in Calvert *et al.* 2016 ^40^ who investigated an under-represented population of patients with Alcohol Use Disorder.

The outcomes measured are summarized in Table 4.

**Table 4.** Overview of measured outcomes in studies on shock.

Study	Sensitivity	Specificity	NPV	PPV	Negative LR	Positive LR	Accuracy	Prevalence	OR	RR	ROC AUC
Ghosh 2017							✓				
Hu 2016											✓
Li 2014	✓	✓					✓				✓
Mahajan 2014							✓				
Mao 2018	✓	✓									✓
Reljin 2018	✓	✓					✓				
Sideris 2016							✓				✓
Blecker 2016	✓			✓							✓
Blecker 2018	✓	✓	✓	✓							✓
Calvert 2016	✓	✓			✓	✓	✓		✓		✓
Donald 2018	✓	✓		✓							✓
Ebrahimzadeh 2018	✓	✓		✓			✓				
Potes 2017	✓	✓	✓	✓		✓					✓
Henry 2015	✓	✓									✓
Strodthoff 2018	✓	✓		✓							

NPV: Negative predictive value. PPV: Positive predictive value. LR: Likelihood ratio. OR: Odds ratio. RR: Risk ratio. ROC AUC: Receiver operating characteristic area under the curve.

***Ongoing studies***. Five studies are currently ongoing, one in Germany ^43^ and the others in the USA ^44– 47^. Two studies are prospective case series ^44, 47^, two studies are prospective cohort studies ^43, 45^ and one is a RCT ^46^. Two of the studies are concerned with developing prediction models, and the others are concerned with implementing machine learning algorithms into clinical practice as early warning systems.

The details of these trials are summarized in Table 5.

**Table 5.** Overview of ongoing studies on shock.

Identifier code	Study Design	Countries and study centers	Hospital setting	Intervention	Sample characteristics	Outcome(s)
NCT03582501	Prospective case series Year of study: 2019–20 Duration: 12 months	USA Mayo Clinic Arizona, Florida & Rochester	NR	Lower body negative pressure to simulate hypovolemia	Estimated: 24 Age: 18–55 Definition: Healthy non-smoker, no history of hypertension, diabetes, CAD and neurologic diseases	Primary outcome Blood pressure Secondary outcome Heart rate
NCT02934971	Prospective cohort study Year of study: 2017–19 Duration: 24 months (up to 6 months follow-up)	Germany, Aachen Aachen University Hospital	Out-patient	Chemotherapy or no chemotherapy	Estimated: 400 Age: ≥ 18 Definition: Patients scheduled for chemotherapy at increased risk of cardiotoxicity and age-matched controls	Primary outcome change in left ventricular ejection fraction
NCT03235193	Prospective cohort study Year of study: 2017 Duration: 3 months	USA, West Virginia Dascena Inc.& University of California	ED, ICU	The InSight algorithm used as an EWS to detect sepsis and severe sepsis detection from EHRs compared to severe sepsis detection from EHRs alone	Estimated: 1241 Age: ≥ 18 Definition: All admitted patients	Primary outcome in-hospital mortality Secondary outcomes length of stay in hospital and ICU, hospital readmission
NCT03644940	RCT Year of study: 2020–21 Duration: 6 months	USA, California Dascena Inc.& University of California	Cardiology, GI, ICU, Medicine, Oncology, Surgery, Transplant and ED	subpopulation- optimized version of InSight compared to the original version used as an early warning system to identify patients at high risk of severe sepsis; followed by physician assessment of sepsis	Estimated n: 51645 Age: >18 Definition: NR	Primary outcomes in-hospital SIRS-based mortality Secondary outcomes in-hospital severe sepsis/ shock-coded mortality; SIRS-based hospital length of stay; Severe sepsis/shock-coded hospital length of stay
NCT03655626	Single-arm trial up to Year of study: 2018–19 up to Duration: 6 months	USA, North Carolina Duke University Hospital	ED	machine learning algorithm to predict sepsis, custom dashboard and monitoring	Estimated n: 3200 Age: >18 Definition: NR	Primary outcome rate of CMS bundle completion for patients with sepsis Secondary outcomes time to sepsis diagnosis; number of patients developing sepsis; number of patients developing sepsis and not treated; length of stay in ED and hospital; inpatient mortality; ICU requirement rate; time from sepsis onset to blood culture, antibiotics, IV fluids, lactate, CMS bundle completion; rate of lactate complete; number of sepsis diagnostic codes per month

USA: United States of America. NR: Not reported. ED: Emergency department. ICU: Intensive care unit. GI: Gastroenterology.

### Respiratory distress/failure

**Figure 2.**
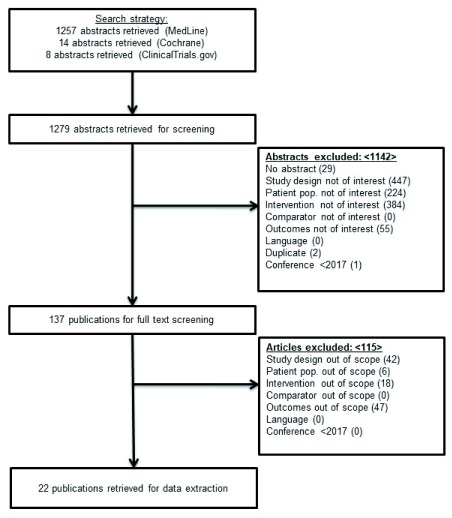
Study selection - Respiratory distress-failure. Pop. = Population.

The search yielded 1279 hits. Screening the titles and abstracts lead to 1142 being excluded. The full texts of the remaining 137 titles were obtained and assessed against the PICOS criteria. Studies were excluded due to irrelevant study design (n=42), population (n=6); intervention (n=18) and outcomes (n=47), and conference proceeding from before 2017 (n=2). A total of 22 studies were finally included in this systematic literature review. None of the trials retrieved from ClinicalTrials.gov were included. The study selection process is depicted in Figure 2.

***Study characteristics***. Of the included studies, 17 were conducted in the US ^33, 48– 63^. Five studies were conducted outside the US; two in Canada ^64, 65^ by the same research group, two in France ^66, 67^ and one in the UK ^68^. In total, 17 studies were retrospective ^33, 48– 50, 52– 55, 58– 66^ and five were prospective ^51, 56, 57, 67, 68^. Of these studies, 12 were single-center ^33, 48, 49, 51, 52, 54, 55, 58, 59, 64– 66^ and 10 studies were multi-center ^50, 53, 56, 57, 60– 63, 67, 68^. Five studies were time-series ^48, 52, 55, 56, 64^, 14 studies were case-series ^33, 49, 51, 53, 54, 57– 62, 65, 66, 68^, one was case-control ^50^ and one was case/time series study ^63^.

The smallest sample of 100 patients came from two single-center retrospective studies ^48, 66^. Ten studies had sample sizes of 101–1000 ^33, 49– 53, 57, 63, 67, 68^; seven studies had sample sizes of 1001–10,000 ^54, 55, 59, 60, 62, 64, 65^; and three had sample sizes larger than 10,000 ^56, 58, 61^. The largest study included more than 50,000 patients admitted to the ED of two centers over a 3-year period ^61^. Several published studies did not report their in-patient setting. When reported, some evaluated data from different wards ^56, 59, 64, 65, 68^, and some included patients admitted only to the ED ^53, 54, 61, 63^, the ICU ^48, 60, 67^ and the surgical ward ^33, 51, 55^.

The characteristics of all published studies are given in Table 6.

**Table 6.** Design aspects of published studies on respiratory distress or failure.

Study	Study Design	Countries and institution(s)	Number of patients (records)	Population/disease definition	In-patient setting
Bejan 2013	Retrospective time series single center	USA, Washington University of Washington	100	NR	ICU
Kumamaru 2016	Retrospective case series single center	USA, Massachusetts Brigham and Women’s Hospital	125	acute pulmonary embolism	NR
Bodduluri 2013	Retrospective case-control multi-center (national data)	USA, Iowa The University of Iowa	153	smokers with or without COPD and non-smokers	NR
Biesiada 2014	Prospective case series single center	USA, Cincinnati Children's Hospital Medical Center & University of Cincinnati	347	current tonsillitis, adenotonsillar hypertrophy or obstructive sleep apnea	Surgery
Reamaroon 2018	Retrospective time series single-center	USA, Michigan University of Michigan	401	mild hypoxia and acute hypoxic respiratory failure	NR
Vinson 2015	Retrospective case series multi-center (4 centers)	USA, California the Kaisers Permanente CREST Network	593	acute pulmonary embolism	ED
Huesch 2018	Retrospective case series single center	USA, Pennsylvania Milton S. Hershey Medical Center	1133	individuals suspected of pulmonary embolism	ED
Mortazavi 2017	Retrospective time series single center	USA, Connecticut Yale University	5214	patients undergoing cardiovascular procedures: CABG, PCI and ICD procedures	Surgery
Pham 2014	Retrospective case series single center	France CHU de Caen, Caen & Hôpital Européen Georges-Pompidou, Paris	NR (100)	individuals suspected of having Venous thromboembolism	NR
Rochefort 2015	Retrospective time series single center	Canada, Quebec McGill University	1649 (2000)	individuals suspected of having Venous thromboembolism	various
Silva 2017	Prospective before-after multi-center (3 centers)	France University Teaching Hospital of Purpan, Toulouse; Hopital Dieu Hospital, Narbonne; Saint Eloi Hospital, Montpellier	136	hemodynamic instability, respiratory failure, multiple trauma, nontraumatic coma, and postoperative complication of abdominal surgery	ICU
Gonzalez 2018	Prospective time series multi-center, multi- national	USA Binham and Women`s Hospital (on behalf of the COPD and ECLIPSE Study investigators)	11655	smokers with or without COPD	various
Tian 2017	Retrospective case series single center	Canada, Quebec Mcgill University	2819 (4000)	individuals suspected of having Venous thromboembolism	various
Choi 2018	Prospective case series multi-center (3 centers)	USA Mayo Clinic, Scottsdale; National Jewish Health, Denve; University of Washington Medical Center, Seattle & Veracyte Inc.	139 (403)	suspected interstitial lung disease	NR
Yu 2014	Retrospective case series single center	USA, Massachusetts Brigham, and Women’s Hospital & Harvard Medical School,	NR (10,330)	individuals suspected of pulmonary embolism	NR
Swartz 2017	Retrospective case series single center	USA, New York New York University & Mount Sinai St. Luke`s Hospital	NR (2400)	individuals suspected of having Venous thromboembolism	various
Liu 2013	Retrospective case series multi-center (21 centers)	USA, California Kaiser Permanente	NR (2466)	NR	ICU
Haug 2013	Retrospective case series multi-center(2 centers)	USA, Utah LDS Hospital and Intermountain Medical Centre	NR (362,924)	NR	ED
Dublin 2013	Retrospective case series multi-center (regional data)	USA, Seattle Group Health Research Institute & University of Washington	NR (5000)	NR	NR
Phillips 2014	Prospective case series multi-center	UK, Llaneli Swansea University, Aberystwyth University & Hywel Dda University Health Board	181	with and without COPD	various
Hu 2016	Retrospective case series single center	USA, Minnesota University of Minnesota	NR (8909)	NR	Surgery
Jones 2018	Retrospective case/time series multi-center (number of centers unknown)	USA, Utah & Washington VA Salt Lake City Health Care System, University of Utah & George Washington University	NR (911)	individuals suspected of pneumonia	ED

NA: Not applicable. NR: Not reported. USA: United States of America. COPD: Chronic obstructive pulmonary disease. ECLIPSE: Evaluations of COPD Longitudinally to Identify Predictive Surrogate Endpoints. UK: United Kingdom. CABG: Coronary artery bypass grafting. PCI: Percutaneous coronary intervention. ICD: Implantable cardioverter defibrillator. ICU: Intensive care unit. ED: Emergency department.

***CDS systems***. About half of the studies developed machine-learning algorithms, whereas the other half focused on natural language processing (NLP) algorithms. One study differed from the rest by developing a computer-aided detection (CAD) system to measure the axial diameter of the right and left pulmonary ventricles, aiding in the diagnosis of pulmonary embolisms ^49^. Many learning algorithms were concerned with detecting pulmonary embolisms and deep vein thrombosis ^53, 54, 58, 59, 64– 67^ as well as pneumonia ^33, 48, 57, 60– 63^. Three studies developed machine-learning algorithms to detect COPD ^50, 56, 69^. One study developed a machine learning algorithm to detect acute respiratory distress syndrome ^52^; while other studies developed machine learning algorithms to detect respiratory distress or failure following a pressure support ventilation trial ^67^, cardiovascular surgery ^55^ and pediatric tonsillectomy ^51^.

The classifiers used in the NLP-based studies were various. However, some commonalities emerged between the studies developing machine-learning algorithms. Multiple studies applied SVM, logistic regression, random forests, K- nearest neighbor (kNN), gradient boosting and neural network models. Various classifiers were explored in 5 studies.

Machine learning and NLP-based algorithms were trained and validated in 20 studies and subsequently tested in an independent dataset in 6 studies ^52, 56, 60– 62, 67^. The CAD system mentioned above and an electronic pulmonary embolism severity index were trained and compared to a reference dataset classified by physicians ^49, 53^.

An overview of the developed learning algorithms is provided in Table 7.

**Table 7.** Overview of the algorithms developed to detect respiratory distress or failure.

		Learning algorithm																				
Study	Predicted disease	NLP	assertion classification	symbolic classifiers	rule or probability based	kNN	ONYX	RF	LR, LASSO penalized	LR, LASSO regularization	LR, not specified	gradient (descent) boosting	Maximum Entropy	SVM	Partial least- squares regression	NegEX	hierarchical classification	Bayesian network	neural network	J48	JRIP	PART
Reamaroon 2018	ARDS							✓			✓			✓								
Gonzalez 2018	COPD, ARDE																		✓			
Bodduluri 2013	COPD					✓																
Phillips 2014	COPD																			✓	✓	✓
Bejan 2013	Pneumonia	✓	✓																			
Dublin 2013	Pneumonia	✓					✓															
Haug 2013	Pneumonia	✓																✓				
Hu 2016	Pneumonia								✓													
Liu 2013	Pneumonia	✓			✓																	
Choi 2018	Pneumonia							✓	✓			✓		✓					✓			
Jones 2018	Pneumonia	✓												✓								
Silva 2017	Postintubation distress														✓							
Mortazavi 2017	Postoperative respiratory failure							✓		✓		✓										
Vinson 2015	Pulmonary embolism				✓																	
Yu 2014	Pulmonary embolism	✓							✓													
Huesch 2018	Pulmonary embolism	✓			✓																	
Kumamaru 2016	Pulmonary embolism *																					
Pham 2014	Pulmonary embolism, DVT	✓											✓									
Rochefort 2015	Pulmonary embolism, DVT													✓								
Swartz 2017	Pulmonary embolism, DVT	✓														✓						
Tian 2017	Pulmonary embolism, DVT	✓		✓																		
Biesiada 2014	Respiratory depression				✓	✓								✓			✓	✓				

*A computer aided detection system was developed for measuring the right ventricular/left ventricular axial diameter ratio and detecting pulmonary embolism. ARDS: Acute respiratory distress syndrome. ARDE: Acute respiratory disease events. COPD: Chronic obstructive pulmonary disease. DVT: Deep vein thrombosis.

One study, Reamoroon *et al.* 2018 ^52^, used a novel sampling technique to accommodate for inter-dependency in longitudinal data. Model accuracy and ROC AUC with this method was <5% better than random sampling and 4–11% better than no sampling.

***Outcome measures***. The majority of the studies reported multiple outcome measures of model performance. The most frequently reported outcome measure was sensitivity, followed by specificity and ROC AUC. Likelihood ratios, on the other hand, were only reported in one study: Silva *et al.* 2017 ^67^ reported eight outcome measures of their novel machine learning model to predict post extubation distress. The outcomes measured across all studies are summarized in Table 8.

**Table 8.** Overview of measured outcomes in studies predicting respiratory distress or failure.

Study	Algorithm	Sensitivity	Specificity	NPV	PPV	negative LR	positive LR	Accuracy	Prevalence	OR	RR	ROC AUC	Diagnostic yield
Kumamaru 2016	CAD							✓				✓	
Bodduluri 2013	ML											✓	
Hu 2016	ML											✓	
Mortazavi 2017	ML											✓	
Rochefort 2015	ML	✓	✓	✓	✓							✓	
Silva 2017	ML	✓	✓	✓	✓	✓	✓	✓				✓	
Vinson 2015	ML	✓	✓	✓	✓			✓					
Biesiada 2014	ML	✓	✓					✓	✓		✓		
Choi 2018	ML	✓	✓									✓	
Gonzalez 2018	ML							✓	✓	✓		✓	
Phillips 2014	ML	✓	✓					✓				✓	
Reamaroon 2018	ML		✓					✓				✓	
Bejan 2013	NLP	✓	✓	✓	✓			✓					
Dublin 2013	NLP	✓	✓	✓	✓								
Haug 2013	NLP											✓	
Liu 2013	NLP	✓	✓	✓	✓								
Pham 2014	NLP	✓			✓								
Swartz 2017	NLP	✓	✓	✓	✓								✓
Tian 2017	NLP	✓	✓	✓	✓								
Yu 2014	NLP			✓	✓							✓	
Huesch 2018	NLP	✓	✓	✓	✓			✓					
Jones 2018	NLP	✓	✓	✓	✓							✓	

NLP: Natural language processing. ML: Machine learning. CAD: Computer aided detection. NPV: Negative predictive value. PPV: Positive predictive value. LR: Likelihood ratio. OR: Odds ratio. RR: Risk ratio. ROC AUC: Receiver operating characteristic area under the curve.

Many of the studies that developed NLP-based algorithms reported negative and positive predictive values, as well as sensitivity and specificity. In contrast, the ROC AUC was the most frequently reported outcome measure of machine learning algorithm performance. It was also the single preferred outcome in three studies ^33, 50, 55^. About half of the studies additionally reported sensitivity, specificity, and accuracy. One study reported specificity with sensitivity set at 90% and 95% to ensure that few disease positive cases were missed ^52^. The single study that developed a CAD system measured the ROC AUC and model accuracy ^49^.

### Infection or sepsis

**Figure 3.**
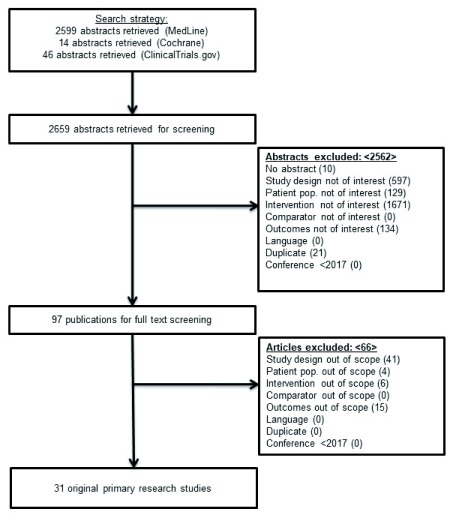
Study selection - infection or sepsis. Pop. = Population.

The search yielded 2659 hits. Screening the titles and abstracts lead to 2562 being excluded. The full texts of the remaining 97 titles were obtained and assessed against the PICOS criteria. Studies were excluded due to irrelevant study design (n=41), population (n=4); intervention (n=6) and outcomes (n=14). A total of 31 studies were finally included in this systematic literature review. Four of these were ongoing trials. The study selection process is depicted in Figure 3.

***Study characteristics***. Of the included studies, 24 were conducted in the US. Three studies were conducted outside the US; one in France; one in the Netherlands and one in the UK. In total, 21 studies were retrospective ^33, 35, 70– 88^ and six were prospective ^89– 94^. There were 21 single-center studies ^33, 70– 75, 77– 83, 86– 88, 90– 92, 94^ and six multi-center studies ^35, 76, 84, 85, 89, 93^. Seven studies were time series ^71, 78, 82, 84– 86, 92^, 18 studies were case series ^33, 35, 70, 72– 76, 80, 81, 83, 87– 91, 93, 94^, one was a case-control ^77^ and one was a matched-controlled study ^79^.

The smallest studies included patients with leukemia ^89^ and combat casualty patients ^90^. Four studies had a sample size below 1000 ^70, 72, 73, 79^, three had a sample size between 1001–10,000 ^33, 71, 87^ and 12 had a sample size larger than 10,000 ^35, 74, 77– 78, 80– 82, 84– 87, 88^. Eight studies had samples even larger than 50,000 ^35, 74, 77, 78, 82, 84, 85, 88^. Large samples were achieved by less restrictive inclusion criteria where all patients admitted to specific ward(s) or hospital(s) over a given time were defined.

Majority of the published studies evaluated data from different wards; several studies included patients admitted only to the ICU ^70, 72, 81, 84– 86, 93^ and surgical ward ^73, 76, 78, 87, 91, 92^, less often the General ward ^33^ and Emergency Department ^74^. Of these, 23 studies included data collected at their own hospital; and four utilized previously collated databases ^76, 81, 84, 86^.

The characteristics of all published studies are given in Table 9.

**Table 9.** Design aspects of published studies on infection or sepsis.

Study	Study Design	Country and institution(s)	Number of patients (records)	Population/disease definition	In-patient setting
Ahmed 2015	Retrospective case series single center	USA, Minnesota Mayo Clinic Rochester	944	NR	ICU
Brasier, 2015	Prospective case series multi-center (3 sites)	USA, Texas Aspergillus Technology Consortium & University of Texas	57	Leukemia	NR
Dente, 2017	Prospective case series single center	USA, Maryland Emory University, Walter Reed National Military Medical Centre	73	Combat casualty patients	NR
Hu, 2016	Retrospective case series single center	USA, Minnesota University of Minnesota	NR (8,909)	NR	General
Konerman, 2017	Retrospective time series single center	USA, Michigan University of Michigan	1,233	Chronic hepatitis c	NR
Legrand, 2013	Prospective case series single center	France, Paris Hôpital Européen Georges Pompidou Assistance Publique- Hopitaux de Paris	202	Infective endocarditis	Surgery
Mani, 2014	Retrospective case series single center	USA, New Mexico University of New Mexico	299	Sepsis	ICU
Mao 2018	Retrospective case series multi-center (5 centers)	USA University of California, Stanford Medical Centre, Oroville Hospital, Bakersfield Heart Hospital, Cape Regional Medical Centre, Beth Israel Deaconess Medical Center	359,390	NR	various
Sanger, 2016	Prospective time series single center	USA, Washington University of Washington	851	Open-abdominal surgery patients	Surgery
Scicluna, 2017	Prospective case series multi-center (2 sites + national database)	Netherlands & UK Amsterdam Academic Medical Center, Utrecht University Medical Center & UK Genomic Advances in Sepsis study	787	Sepsis	ICU
Sohn, 2016	Retrospective case series single center	USA, Minnesota Mayo Clinic Rochester	751	Colorectal surgery patients	Surgery
Taylor, 2018	Retrospective case series single center	USA, Connecticut Yale University School of Medicine,	55,365 (80,387)	Suspected urine tract infection	ED
Hernandez 2017	Retrospective case series single center	UK, London Imperial College Healthcare NHS Trust	> 500,000	NR	NR
Bartz-Kurycki 2018	Retrospective case series multi-center (national database)	USA, Texas University of Texas	13,589	NR	Surgery
Beeler 2018	Retrospective case-control single center	USA, Indiana Indiana University Health Academic Health Center	NR (70,218)	Central venous line with or without central line- associated bloodstream infections	NR
Bihorac 2018	Retrospective time series single center	USA, Florida University of Florida Health	51,457	NR	Surgery
Chen 2018	Retrospective matched pairs (1:1 case matching) single center	USA, Kansas University of Kansas Health System	358	Stage 3 AKI and non-AKI controls	NR
Cheng 2017	Retrospective case series single center	USA, Kansas University of Kansas Medical Center	33,703 (48,955)	NR	NR
Desautels 2016	Retrospective case series single center	USA, California Dascena Inc.& University of California	NR (21,176)	NR	ICU
Koyner 2015	Retrospective time series single center	USA, Chicago University of Chicago	NR (121,158)	NR	NR
LaBarbera 2015	Retrospective case series single center	USA, Pennsylvania Pinnacle Health Hospital, Harrisburg	198	Clostridium difficile infection	NR
Mohamadlou 2018	Retrospective time series multi-center (2 sites)	USA Dascena Inc., University of California & Stanford University	68,319	NR	ICU
Nemati 2018	Retrospective time series multi-center (3 sites)	USA, Georgia Emory University School of Medicine & Georgia Institute of Technology	69,938	NR	ICU
Parreco 2018	Retrospective time series single center	USA, Florida University of Miami	NA (22,201)	NA	ICU
Taneja 2017	Prospective case series single center	USA, Illinois University of Illinois	444	Suspected sepsis	NR
Weller 2018	Retrospective case series single center	USA, Minnesota Mayo Clinic Rochester	1,283	Colorectal surgery patients	Surgery
Wiens 2014	Retrospective case series single center	USA single center not specified	NR (69,568)	NR	various

NA: Not applicable. NR: Not reported. USA: United States of America. UK: United Kingdom. ICU: Intensive care unit. ED: Emergency department. AKI: Acute kidney injury.

***CDS systems***. The machine learning algorithms evaluated in the studies were developed to predict a range of diseases. These included sepsis ^33, 35, 72, 78, 81, 85, 93, 94^, acute kidney injury ^70, 78– 80, 82, 84, 91^, surgical site infections ^33, 73, 76, 87, 92^, central line-associated bloodstream infections ^77, 86^, *Clostridium difficile* ^83, 88^, pulmonary *aspergillosis* ^89^, bacteremia ^90^, fibrosis ^71^, urine tract infection ^33, 74^ and infections in general ^75^.

Almost half of the studies compared different machine learning algorithms, while the others focused only on Bayesian algorithms ^73, 92^, decision tree algorithms ^84^, ensemble algorithms ^35, 71, 82, 83, 90, 93^, regression algorithms ^33, 78, 85^, regularization algorithms ^81, 88^ and rule learning ^70^. The most frequently applied model was random forest (15 studies) followed by logistic regression (10 studies), support vector machines (5 studies), naïve Bayes (5 studies) and gradient tree boosting (5 studies).

One study compared three different sampling methods for handling class imbalance; under-sampling the majority class (RANDu), over-sampling the minority class (RANDo) and synthetic minority over-sampling (SMOTE). This was a very large study including more than 500,000 patients to predict the onset of infections ^75^. The authors found that SMOTE outperformed the other techniques and improved model sensitivity. Two other very large studies used the RANDu method ^80^ and mini-batch stochastic gradient descent with backpropagation ^85^. No other studies were concerned with imbalance in disease positive and negative classification.

Machine learning models were trained and validated in 26 studies and subsequently tested in an independent dataset in four studies ^35, 72, 75, 77^.

The machine learning algorithms used are illustrated in Table 10.

**Table 10.** Overview of machine learning algorithms evaluated in studies on infection or sepsis.

		Machine learning algorithm																											
Study	Predicted disease	Rule learning	NB	tree augmented NB	AODE	lazy Bayesian rules	Bayesian GLM	Bayesian network analysis	CART	decision tree classifier	neural network	RF	(extreme) gradient boosting	adaptive boosting	ensemble classifier	k nearest neighbor	MARS	GPS	Laaso penalized LR	LR, not specified	SVM	generalized additive model	GLM	stepwise regression	polynomial linear model	ploynomial spline regression	Weibull PH model	L2-regularised LR	elastic net regularization
Ahmed 2015	AKI	✓																											
Legrand, 2013	AKI						✓				✓	✓	✓									✓	✓	✓	✓	✓			✓
Cheng 2017	AKI											✓		✓						✓									
Koyner 2015	AKI												✓																
Bihorac 2018	AKI, sepsis																					✓							
Mohamadlou 2018	AKI, Stage 2/3									✓																			
Chen 2018	AKI, Stage 3									✓	✓	✓			✓	✓													
Dente, 2017	bacteremia											✓																	
Beeler 2018	CLABSI											✓								✓									
Parreco 2018	CLABSI										✓		✓						✓										
LaBarbera 2015	clostridium difficile											✓																	
Wiens 2014	clostridium difficile																											✓	
Konerman, 2017	fibrosis											✓																	
Hernandez 2017	infection		✓							✓		✓									✓								
Brasier, 2015	pulmonary aspergillosis								✓			✓					✓	✓											
Mani, 2014	sepsis		✓	✓	✓	✓						✓				✓				✓	✓								
Mao, 2018	sepsis												✓																
Scicluna, 2017	sepsis											✓																	
Desautels 2016	sepsis																												✓
Nemati 2018	sepsis																										✓		
Taneja 2017	sepsis		✓									✓		✓					✓		✓								
Sanger, 2016	SSI		✓																	✓									
Sohn, 2016	SSI							✓																					
Bartz-Kurycki 2018	SSI											✓								✓									
Weller 2018	SSI		✓									✓		✓					✓		✓								
Hu 2016	SSI, UTI, pneumonia, sepsis																		✓										
Taylor, 2018	UTI										✓	✓	✓	✓						✓	✓								✓

AKI: Acute kidney injury. SSI: Surgical site infection. UTI: Urinary tract infections. CLABSI: Central line-associated bloodstream infections. NB: Naive Bayes. AODE: Averaged one dependence estimators. CART: Classification and regression tree. RF: Random forest. MARS: Multivariate Adaptive Regression Splines GPS: Generalized path seeker algorithm. LR: Logistic regression. SVM: Support vector machine. GLM: Generalized linear model. PH: Proportional hazards.

***Outcome measures***. The most frequently reported outcome measure was the ROC AUC. Three studies did not report this measure: Ahmed *et al.* 2015 ^70^ developed an algorithm based on decision rules; Legrand *et al.* 2013 ^91^ was primarily interested in identifying risk factors of AKI after cardiac surgery; and Scicluna *et al.* 2017 ^93^ was primarily concerned with identifying genetic biomarkers of sepsis.

Sensitivity and specificity were reported together in 14 studies ^35, 70– 72, 74, 75, 78, 81– 84, 87, 90, 92^. When specificity was not reported, sensitivity was reported together with PPV; and when sensitivity was not reported, this was due to sensitivity being set at a fixed value to report other diagnostic performance measures. In relation to the prior observation, more studies reported PPV than NPV. Four studies reporting likelihood ratios reported both negative and positive likelihood ratios ^70, 74, 81, 84^.

An overview of measured outcomes is illustrated in Table 11.

**Table 11.** Overview of measured outcomes in studies predicting sepsis or infection.

Study	Sensitivity	Specificity	NPV	PPV	negative LR	positive LR	Accuracy	Prevalence	OR	RR	ROC AUC
Ahmed 2015	✓	✓	✓	✓	✓	✓			✓		
Brasier, 2015							✓				✓
Dente, 2017	✓	✓					✓				✓
Hu, 2016											✓
Konerman, 2017	✓	✓	✓	✓				✓			✓
Legrand, 2013									✓		
Mani, 2014	✓	✓	✓	✓							✓
Mao 2018	✓	✓	✓	✓			✓				✓
Sanger, 2016	✓	✓	✓	✓			✓				✓
Scicluna, 2017								✓			
Sohn, 2016											✓
Taylor, 2018	✓	✓			✓	✓	✓				✓
Hernandez 2017	✓	✓									✓
Bartz-Kurycki 2018											✓
Beeler 2018											✓
Bihorac 2018	✓	✓	✓	✓			✓	✓		✓	✓
Chen 2018	✓			✓							✓
Cheng 2017	✓			✓							✓
Desautels 2016	✓	✓			✓	✓	✓		✓		✓
Koyner 2015	✓	✓	✓	✓							✓
LaBarbera 2015	✓	✓		✓							✓
Mohamadlou 2018	✓	✓			✓	✓	✓		✓		✓
Nemati 2018		✓					✓				✓
Parreco 2018	✓	✓	✓	✓			✓				✓
Taneja 2017											✓
Weller 2018											✓
Wiens 2014	✓			✓							✓

NPV: Negative predictive value. PPV: Positive predictive value. LR: Likelihood ratio. OR: Odds ratio, RR: Risks ratio. ROC AUC: Receiver operator curve area under the curve.

***Ongoing studies***. Four trials are currently ongoing, one in Germany and the others in the USA, all concerned with the prediction of sepsis. Three of them are prospective studies and one is retrospective. The retrospective study aims to develop a prediction algorithm based on claims data, EHRs, risk factors and survey data of an estimated 50,000 adult patients admitted to the ED. The German study NCT03661450 ^95^ is a single-arm trial evaluating the utility of a CDS system to identify SIRS or sepsis from EHRs in a pediatric ICU population. Another single-arm trial NCT03655626 ^47^ is concerned with implementing a sepsis prediction algorithm in clinical practice as an early warning system. NCT03644940 ^46^ is comparing two versions of InSight introduced into clinical practice as an early warning system.

## Discussion and conclusions

This systematic literature review shows that over the last 2 decades, there has been an increased interest in CDS as means of supporting clinicians in acute care. CDS has been investigated for several applications ranging from the detection of health conditions ^60, 61^, to the prediction of deterioration or adverse events ^40, 55, 76, 81, 83, 84^. Applications also include therapy guidance, as well as updating clinicians on new or changed recommendations ^96^. CDS can also provide guidance by predicting clinical trajectories for different patient profiles over time ^97^.

From rule-based algorithms and simple regression models, CDS has evolved to encompass a multitude of techniques in Machine-Learning ^98^. These techniques can be dependent on the problem selected and the data types used. Across the three disease areas investigated, the frequent use of random forest classifiers (28.1%), support vector machines (21.9%), boosting techniques (20.3%), LASSO regression (18.8%) and unspecified logistic regression models (10.9%) were observed. The use of more complex modeling such as maximum entropy, Hidden Markov Models (for temporal data analysis) as well as Convolutional Neural Networks has also emerged over the last few years. In the respiratory distress area, the use of NLP models is more common as radiology reports and clinical notes are the main source of input. Different image analysis techniques have been developed to aid in the prediction and diagnosis of respiratory events from radiology images.

Typical measures of NLP model performance include sensitivity, specificity and predictive values. In measuring ML algorithm performance, sensitivity, specificity and ROC AUC are more common. A wide range of outcome measure were reported in research on less-investigated health conditions ^40, 67^; and also when uncommon, more complex algorithms were compared to basic algorithms ^74, 78, 81, 84^. This is not surprising given the novelty of these applications.

Many of the ML algorithms and all of the NLP models covered in this work were based on medical data collected in certain clinical sites rather than publicly available data. Datasets from national audits, completed studies or other online sources can additionally play a role, particularly in model validation and testing. This could aid in the adoption and wider use of CDS systems. In this SLR, publicly available datasets were mainly utilized for developing prediction models of heart arrhythmias ^29– 31^, hypotension ^32^, septic shock ^28, 33, 40, 41^, COPD ^50^, pneumonia ^33^ and a range of infections ^33, 76, 78, 81, 84, 86^. In only three cases were they used for testing model performance in sepsis and septic shock prediction; this included the Insight algorithm ^35, 85, 93^.

Most of the studies identified in this SLR were retrospective and originated in the USA where electronic health records (EHR) are commonly used. This makes it easier to access and compile large amounts of patient-level information. Many of the studies on shock and infection/sepsis based their models on data extracted from EHRs and utilized large sample sizes. The diversity in the identified CDS systems makes it challenging to draw conclusions on methodology. The lack of comparisons between different classifiers within studies, especially for the indication of shock, adds to this challenge. To assess the effectiveness of ML algorithms, future research should evaluate multiple algorithms on standard well-labeled datasets.

Class imbalance can be an important issue when training classifiers on datasets for the conditions highlighted in this work. Unequal distributions can arise naturally between disease negative and positive classes when forming validation sets, particularly when disease prevalence is low ^75^. We refer the reader to several machine learning reviews that have addressed this issue ^99– 101^. Another important issue in forming disease positive classes relates to the analysis of repeated-measures within subjects, for example, when clinical records are available for each hospitalization day. Several studies have approached this by selecting the first record indicating positive for a health condition. Few researchers have utilized all records and corrected for within-subject variation. An example is the selection of cases depending on observed correlation decay ^52^.

In all three areas investigated, the number of retrospective studies exceeded by far the number of prospective studies conducted in a clinical setting. This highlights the challenges in substantiating clinical performance while bringing new clinical decision tools to routine in-hospital patientcare. Examples of algorithms that can be integrated in clinical practice include InSight ^45, 46^ and Sepsis Watch ^47^ which are intended for predicting sepsis and septic shock.

The current systematic literature review did not search multiple bibliographic databases or clinical trial registers; and focused on diagnostic performance rather than other outcomes. In fact, during study screening, trials that evaluated the impact of early warning systems on measures of clinical workflow, rate of re-admissions and/or mortality were discarded as they are somehow out of the focus of this work. This implies that there may be more CDS systems used in practice for the three populations investigated within this research, where the outcomes measured are different. Limiting the search to publications in English and to studies conducted in particular countries; and the exclusion of study protocols identified from the bibliographic database search without checking for later publications from the same authors may have further limited the studies selected. Nevertheless, studies identified within each population represented a diverse range of models applied in different hospital settings trained to predict a range of health conditions. The most widely researched conditions were sepsis and septic shock, venous thromboembolisms, acute kidney injury and surgical site infections.

Specific challenges were identified in collecting sufficient data for training CDS systems on hemodynamic instability. Patients who are, for example, at risk of hemorrhage due to a traumatic injury need to be carefully monitored; and the speed by which they reach a critical state may influence data and study management. It may also be difficult to find healthy volunteers who are willing to undergo procedures like lower body negative pressure which can be unpleasant ^36^. Identification of cases in need of hemodynamic interventions can lend towards larger sample size ^19^. Other conditions that need further attention are clostridium difficile and CLABSI. Prediction models were driven by almost perfect specificity and very low (<10%) sensitivity ^77, 83, 86, 88^. Considering that these studies used a wide range of features from the EHRs and a large number of patients, except LaBarbera, Nikiforov ^83^, there is a need to better understand the risk factors to improve sensitivity.

Based on the literature reviewed in this work, as well as several recent surveys and workshops, we would recommend the following points to be addressed when bringing a new CDS tool to critical care ^14, 102– 104^:

- Integrating CDS in clinical workflows without adding unnecessary extra work to busy clinical teams. The CDS101 toolbox by HIMMS highlights the “CDS five rights”, which are certainly applicable to critical care ^105^: Providing the right information in the right intervention format, to the right person at the right point in their workflow, and through the right channel.
- Developing tools and concrete proof-points able to assess CDS efficacy in the clinic. This also highlights the importance of providing continuous feedback to clinicians.
- The importance of easy to use user interfaces and focusing on human-computer interaction during deployment.
- Efficient training that is available when needed.
- Being aware of alert or alarm fatigue and not overloading clinicians with alerts due to CDS. The intensive care unit is already plagued with alarms, and if anything, CDS should help in reducing alarms by bundling alerts according to underlying conditions.
- Displaying the rationale for decisions as well as the underlying data to clinical users would lead to improved adoption.
- Understanding ethical challenges for CDS, as well as a careful risk assessment in every site before deployment ^106^.
- Being able to repeat/standardize implementation across organizations – most prospective studies reviewed in this work covered single centers. Only a few were multi-center studies.

## Data availability

### Underlying data

All data underlying the results are available as part of the article and no additional source data are required

### Extended data

Figshare: Evidence-based Clinical Decision Support Systems for the prediction and detection of three disease states in critical care: A systematic literature review. Extended data - Table 1-Search strategy for shock (hemodynamic (in-stability) in MEDLINE.docx. https://doi.org/10.6084/m9.figshare.9892109.v1 ^25^.

Figshare: Working title: Evidence-based Clinical Decision Support Systems for the prediction and detection of three disease states in critical care: A systematic literature review. Extended data - Table 2-Search strategy for respiratory distress or respiratory failure in MEDLINE.docx. https://doi.org/10.6084/m9.figshare.9892112.v1 ^26^.

Figshare: Working title: Evidence-based Clinical Decision Support Systems for the prediction and detection of three disease states in critical care: A systematic literature review. Extended data - Table 3-Search strategy for infection or sepsis in MEDLINE.docx. https://doi.org/10.6084/m9.figshare.9892115.v1 ^27^.

### Reporting guidelines

Figshare: PRISMA checklist for ‘Evidence-based Clinical Decision Support Systems for the prediction and detection of three disease states in critical care: A systematic literature review’. https://doi.org/10.6084/m9.figshare.9894107.v1 ^107^.

Data are available under the terms of the Creative Commons Zero “No rights reserved” data waiver (CC0 1.0 Public domain dedication).
