# Supplementary material for: Evidence-based Clinical Decision Support Systems for the prediction and detection of three disease states in critical care: A systematic literature review
Source: F1000Res. 2019 Nov 27;8:1728. Originally published 2019 Oct 8. [Version 2] doi: 10.12688/f1000research.20498.2 (PMC6894361; doi:10.12688/f1000research.20498.2)
Supplement: Peer review history

# Peer review: Reviewer response for version 2

**DOI:** 10.5256/f1000research.23644.r57156

**Published:** 2019-12-04

**Authors:** Stavros Nikolakopoulos

**Affiliations:** 1 Department of Biostatistics & Research Support, Julius Center for Health Sciences and Primary Care, University Medical Center Utrecht, Utrecht, The Netherlands

My comments were adequately addressed in the text. No further comments.

I confirm that I have read this submission and believe that I have an appropriate level of expertise to confirm that it is of an acceptable scientific standard.

# Peer review: Reviewer response for version 2

**DOI:** 10.5256/f1000research.23644.r57155

**Published:** 2019-11-28

**Authors:** Milena Kovacevic

**Affiliations:** 1 Department of Pharmacokinetics and Clinical Pharmacy, Faculty of Pharmacy, University of Belgrade, Belgrade, Serbia

Thank you for addressing my comments. I have no further comments to make.

I confirm that I have read this submission and believe that I have an appropriate level of expertise to confirm that it is of an acceptable scientific standard.

# Peer review: Reviewer response for version 1

**DOI:** 10.5256/f1000research.22530.r56200

**Published:** 2019-11-18

**Authors:** Milena Kovacevic

**Affiliations:** 1 Department of Pharmacokinetics and Clinical Pharmacy, Faculty of Pharmacy, University of Belgrade, Belgrade, Serbia

The review summarizes the utilization of clinical decision support (CDS) systems in three selected states in critical care – shock/hemodynamic (in-)stability; respiratory distress/failure; and infection/sepsis. The background of the study has a strong rationale.

 The study comprised the results from primary sources, describing models/algorithms used to detect and alert clinicians to the presence of these conditions, as well as models/algorithms developed to predict deterioration in an individual patient state, leading to these selected conditions.

 The systematic review was performed and the findings are presented in line with the PRISMA guidelines. Variables for which data were sought were clearly stated (PICOS) in Table 1.

*Specific comments:*

- What I found especially beneficial for the readers and future research in this area, is Table 2 with the presented collected data used for training algorithms. 
- It would be beneficial to provide additional information whether an internal or external validation was performed - within Table 4 (measured outcomes in studies on shock), Table 8 (measured outcomes in studies on respiratory distress/failure) and Table 11 (measured outcomes in studies on infection/sepsis).
- What was the rationale for including the studies predicting acute kidney injury within the Infection/sepsis results section? If it is about the decline in glomerular filtration rate due to hypotension seen in sepsis, it might have been presented within the Shock section.
- Table 7: include the abbreviations for ARDS (Acute respiratory distress syndrome), ARDE (Acute respiratory disease events) and DVT (deep vein thrombosis) below the Table.
- Table 9: include the abbreviation for AKI (Acute kidney injury) below the Table.

I confirm that I have read this submission and believe that I have an appropriate level of expertise to confirm that it is of an acceptable scientific standard.

# Peer review: Reviewer response for version 1

**DOI:** 10.5256/f1000research.22530.r56199

**Published:** 2019-11-06

**Authors:** Stavros Nikolakopoulos

**Affiliations:** 1 Department of Biostatistics & Research Support, Julius Center for Health Sciences and Primary Care, University Medical Center Utrecht, Utrecht, The Netherlands

The authors report on a systematic review in order to assess the state-of-the -art in the field of Clinical Decision Support (CDS) systems in the last 5 years (2013-2018). They review and report on study designs, outcomes and methods employed in CDS in the scientific literature as well as in study databases (like Clinicaltrials.gov).

 The paper is clearly written and organized. The methodology for the systematic review is solid and comprehensive. The topic is also very relevant and timely. I do have some concerns which are mentioned below:

- The authors could potentially include in the study (as described by the inclusion criteria), conference abstracts that were published only as abstracts in 2017 or 2018, even without subsequent publication. I assume they do that in order to somehow keep up with later developments even if they are not published elsewhere, given the very fast pace of the research area. However, they exclude protocols of studies that were published in the same (or more extended) time frame, which seems slightly inconsistent. Some discussion concerning this choice would be enlightening.
- There seems to be some confusion with terminology, with unknown consequences on the review's results. The authors seem to separate "machine learning" methods, from "statistical" methods ( Table 1: "Multivariable hierarchal logistic regression models*** (models which are based only on statistics - but there is no machine learning)", as an exclusion criterion ). This is clearly not the suitable platform to resolve this issue, but, the distinction between machine learning and statistics is not at all that clear. Specifically, under the term "supervised learning", any regression method (statistics) could be classified. So, logistic regression IS a machine learning method. So is LASSO and several other methods reported. Again, this is not the appropriate place for going into further details, but there is certainly some confusion, especially when in the results Logistic regression keeps appearing as a preferred method. 
- Again concerning terminology, the term "accuracy" appears often in the results section. Sometimes it is reported as a different outcome than i.e. ROC AUC, sensitivity and specificity. All the latter methods are quantifying "accuracy" in some way and some clarification is needed. 

**Minor comments:**

- Table 1: Treatment/Intervention, a parenthesis is missing.
- Tables 7 & 10: Maybe reverse the orientation of the column titles, it is impossible to read on a screen.

I confirm that I have read this submission and believe that I have an appropriate level of expertise to confirm that it is of an acceptable scientific standard, however I have significant reservations, as outlined above.
